# New insights into intrinsic foot muscle morphology and composition using ultra‐high‐field (7-Tesla) magnetic resonance imaging

**DOI:** 10.1186/s12891-020-03926-7

**Published:** 2021-01-21

**Authors:** Melinda M. Franettovich Smith, James M. Elliott, Aiman Al-Najjar, Kenneth A. Weber, Mark A. Hoggarth, Bill Vicenzino, Paul W. Hodges, Natalie J. Collins

**Affiliations:** 1 School of Health and Rehabilitation Sciences, The University of Queensland, 4072 Brisbane, QLD Australia; 2 Faculty of Medicine and Health, The Kolling Research Institute, The University of Sydney, the Northern Sydney Local Health District, 2006 Sydney, New South Wales Australia; 3 Department of Physical Therapy and Human Movement Sciences, Northwestern University, Chicago, IL USA; 4 Centre for Advanced Imaging, The University of Queensland, 4072 Brisbane, QLD Australia; 5 Systems Neuroscience and Pain Lab, Division of Pain Medicine, Department of Anesthesiology, Perioperative and Pain Medicine, Stanford University School of Medicine, Palo Alto, CA USA; 6 La Trobe Sport and Exercise Medicine Research Centre, School of Allied Health, Human Services and Sport, College of Science, Health and Engineering, La Trobe University, 3086 Melbourne, Australia

**Keywords:** Magnetic resonance imaging, Foot, Muscle fat infiltration, Plantar intrinsic muscles, Foot core, Morphology, Musculoskeletal imaging, Muscle segmentation

## Abstract

**Background:**

The intrinsic muscles of the foot are key contributors to foot function and are important to evaluate in lower limb disorders. Magnetic resonance imaging (MRI), provides a non-invasive option to measure muscle morphology and composition, which are primary determinants of muscle function. Ultra-high-field (7-T) magnetic resonance imaging provides sufficient signal to evaluate the morphology of the intrinsic foot muscles, and, when combined with chemical-shift sequences, measures of muscle composition can be obtained. Here we aim to provide a proof-of-concept method for measuring intrinsic foot muscle morphology and composition with high-field MRI.

**Methods:**

One healthy female (age 39 years, mass 65 kg, height 1.73 m) underwent MRI. A T1-weighted VIBE – radio-frequency spoiled 3D steady state GRE – sequence of the whole foot was acquired on a Siemens 7T MAGNETOM scanner, as well as a 3T MAGNETOM Prisma scanner for comparison. A high-resolution fat/water separation image was also acquired using a 3D 2-point DIXON sequence at 7T. Coronal plane images from 3T and 7T scanners were compared. Using 3D Slicer software, regions of interest were manually contoured for each muscle on 7T images. Muscle volumes and percentage of muscle fat infiltration were calculated (muscle fat infiltration % = Fat/(Fat + Water) x100) for each muscle.

**Results:**

Compared to the 3T images, the 7T images provided superior resolution, particularly at the forefoot, to facilitate segmentation of individual muscles. Muscle volumes ranged from 1.5 cm^3^ and 19.8 cm^3^, and percentage muscle fat infiltration ranged from 9.2–15.0%.

**Conclusions:**

This proof-of-concept study demonstrates a feasible method of quantifying muscle morphology and composition for individual intrinsic foot muscles using advanced high-field MRI techniques. This method can be used in future studies to better understand intrinsic foot muscle morphology and composition in healthy individuals, as well as those with lower disorders.

## Background

The intrinsic foot muscles are those that have their anatomical attachments within the foot, in contrast to the extrinsic muscles that originate on the leg and insert on the foot. Together with passive structures (such as the bony arches, fat pads, ligaments and plantar fascia), the intrinsic foot muscles assist attenuation of forces associated with the foot-ground collision and stiffening of the foot for propulsion [1, 2]. The distinguishing contribution of the intrinsic foot muscles, compared to passive structures, is the ability to modulate the energetic function of the foot to respond to changing demands (e.g. acceleration and deceleration, surfaces and footwear) [1, 3].

The important contribution of the intrinsic foot muscles to foot function suggests they should be considered when evaluating and treating patients with lower limb disorders. The challenge is to measure these muscles in a valid manner in clinical and research settings [4]. Measures of muscle strength cannot distinguish contributions from intrinsic and extrinsic muscles. The anatomical configuration of the intrinsic foot muscles, such as their small size and depth within the foot, limits electromyography studies to invasive intramuscular evaluations. Imaging modalities, such as ultrasound imaging (US) and magnetic resonance imaging (MRI), provide a non-invasive option to measure muscle morphology (size and shape) and composition, which are primary determinants of muscle function (force output) [5]. Three-dimensional MRI is considered a gold standard for quantification of muscle morphology as it allows evaluation of the whole muscle (i.e. volume). This contrasts ultrasound, which typically involves 2-dimensional imaging and measures of cross-sectional area or thickness from transverse and longitudinal views. MRI also permits quantification of muscle composition (e.g. fat infiltration), which can also affect the force producing capacity of a muscle [6].

MRI of muscle of the foot has been used to evaluate intrinsic foot muscle morphology in several patient populations, such as individuals with diabetes [7–17], plantar heel pain [18, 19], foot pain [20, 21], Charcot-Marie-Tooth [22, 23], and chronic ankle instability [24, 25], as well as to evaluate the effect of interventions such as physical therapy [24], footwear [26, 27] and foot exercise [28–30]. Table 1 provides a summary of published methods from studies that have evaluated intrinsic foot muscle morphology and composition. Previous studies have used a variety of methods, that vary in terms of field strengths from 0.5 T (0.5T) to 3T and acquisition techniques (e.g. T1-, T2- and proton-density-weighted; gradient echo, spin echo and other sequences).

**Table 1:** A non-systematic summary of methods across studies that describe intrinsic foot muscle (IFM) morphology using magnetic resonance imaging (MRI)

Citation	Reliability	MRI sequence	Slice selection	Muscle of interest	ROI selection	Fat detection	Measure
Andersen et al., 2004 [7]	Not reported	1T; T1-weighted spin echo; 4 mm slice thickness; slice interval 10 mm	All slices, the first section being randomly placed within the first interslice 10-mm interval	All, as a single group	Semi-automated user set pixel intensity threshold, stereological point-counting method	N/A	Muscle volume (expressed as a percentage of the value of the matched control participant)
Andreassen et al., 2009 [8]	Not reported	1.5T; T1-weighted fast spin echo; 4 mm slice thickness	All slices	All, as a single group	Semi-automated user set pixel intensity threshold, stereological point-counting method	N/A	Muscle volume (cm^3^)
Brash et al., 1999 [9]	Not reported	0.5T; T1-weighted gradient-echo and T2-weighted spin echo	Sagittal section through first metatarsal head	Muscle tissue under the first metatarsal head	Not described	N/A	Percentage of CSA that was magnetization transfer active
Bus et al., 2002 [12]	Not reported	3T; T2-weighted fast spin echo; 3 mm slice thickness; 0.15 mm interslice gap; acquisition time 30 min	2 datasets collected in frontal plane; 1st dataset 40–46 slices from mid-tarsal joint proximally & distal IP of 2nd toe distally; 2nd dataset 6 slices from distal metatarsal region; one slice through head 5th metatarsal selected for quantitative analysis	All, as a single group	Semi-automated signal intensity levels using CCHIPS software, verified by visual inspection	N/A	Muscle CSA (expressed as a percentage of total foot CSA)
Bus et al., 2006 [10]	Intra-rater reliability; 4 weeks between measures; weighted kappa = 0.94	1.5T; T1-weighted spin echo; slice thickness 3 mm; 0.9 mm interslice gap	Sagittal plane images oriented parallel to long axis 2nd metatarsal and perpendicular to sole of the foot − 19 slices acquired between 1st and 5th metatarsal heads; coronal plane images oriented perpendicular to sagittal plane images − 20 slices between proximal phalanx and cuneiforms; one slice through head 5th metatarsal selected for analysis	All, as a single group	Visual inspection	Semi-quantitative 5-point scale	Fatty atrophy: 0 = healthy muscle or no atrophy; 1 = mild atrophy; 2 = moderate atrophy; 3 = severe atrophy; 4 = almost no or no muscle tissue visible
Bus et al., 2009 [11]	Cited Bus et al., 2006 *(Intra-rater reliability;* *weighted kappa = 0.94)*	1.5T; T1-weighted spin echo; slice thickness 3 mm; 0.9 mm interslice gap	Sagittal plane images − 19 slices acquired between 1st and 5th metatarsal heads; coronal plane images − 20 slices between proximal phalanx and cuneiforms; one slice through head 5th metatarsal selected for analysis	All, as a single group	Visual inspection	Semi-quantitative 5-point scale	Fatty atrophy: 0 = healthy muscle tissue or no atrophy; 1 = mild atrophy; 2 = moderate atrophy; 3 = severe atrophy; 4 = almost complete or complete loss of muscle tissue
Chang et al., 2012 [18]	Intra-rater reliability; one image processed 5 times; coefficient of variation of muscle CSA = 1.3%	1.5T; T1-weighted spin echo; slice thickness 4 mm; contiguous slices; acquisition time 25 min	Frontal plane images acquired perpendicular to plantar aspect of foot; every image from calcaneus through to image containing maximum diameter of sesamoid bones	All, as a single group (but excluding EDB)	Interactive custom software programmed in Matlab; Semi-automated user set pixel intensity threshold	N/A	Muscle volume (cm^3^)
Chen et al., 2016 [26]	Cited Cheung et al., 2016 (*who cited Chang 2012)*	1.5T; T1-weighted spin echo; slice thickness 4 mm; contiguous slices	Sagittal and frontal plane acquisition; entire length of foot	All, as a single group	Segmented by excluding all non-contractile tissues in Mimics software	N/A	Muscle volume (mm^3^/kg)
Cheung et al., 2016 [19]	Cited Chang et al., 2012	1.5T; T1-weighted; Slice thickness 4 mm; contiguous slices	Images acquired perpendicularly to the plantar aspect of the foot; entire length of foot	All, as a single group	Segmented by excluding all non-contractile tissues in Mimics software	N/A	Muscle volume (mm^3^/kg)
Cheuy et al., 2013a [13]	Inter- and intra-rater reliability; 2 raters processed 46 slices with at least 14 days between measures; all ICCs > 0.9	3T; optimised to fat; spin echo pulse; slice thickness 3.5 mm; acquisition time 9–12 min;	Coronal plane images acquired; 35–65 slices; the forefoot (mid-metatarsal), the midfoot (tarsometatarsal joint of the second metatarsal), the hindfoot (talonavicular joint)	Plantar side muscles, as a single group	Signal intensity threshold automatically identified with optional manual editing of borders and thresholds as required, Matlab software	Quantitative	Volume (cm^3^): Subcutaneous fat; Lean muscle; Intermuscular adipose tissue
Cheuy et al., 2013b [14]	Not reported	3T; spin echo pulse; slice thickness 3.5 mm; acquisition time 12 min	Coronal plane images acquired; 35 slices; talonavicular joint to tarsometatarsal joint	Intrinsic foot muscles between the talonavicular and tarsometatarsal joints	Signal intensity threshold automatically identified with optional manual editing of borders and thresholds as required, Matlab software	Quantitative	Volume (cm^3^): Subcutaneous fat; Lean muscle; Intermuscular adipose tissue; Intrinsic foot muscle ratio (ratio of intermuscular volume to lean muscle volume
Feger et al., 2016 [25]	Not reported	3T; spiral gradient echo; slice thickness 5 mm; acquisition time 15 min	Axial slices; entire foot	ABH; ADDH-O; ADDH-T; FHB; ABDM; FDM; EDB; FDB; QP, Interosseous	Manual segmentation of each muscle perimeter on each slice using custom software written in MatLab	N/A	Muscle volume (cm^3^/m.kg)
Feger et al., 2019 [24]	Cited Handsfield et al., 2014 (*study of leg muscle segmentation; inter-user variability reported as acceptable at < 0.6%*)	3T; spiral gradient echo; slice thickness 5 mm; acquisition time 15 min	Axial slices were obtained in sets of 20 contiguous images from just posterior to the calcaneus anteriorly through the entire foot.	ABH; ADDH-O; ADDH-T; FHB; ABDM; FDM; EDB; FDB; QP, Interosseous	Manual segmentation of each muscle perimeter on each slice using custom software written in MatLab	N/A	Muscle volume (cm^3^/m.kg)
Gallardo et al., 2006 [23]	Not reported	1.5T; T1-weighted fast spin-echo and fat-supressed proton density-T2 weighted fast spin-echo in both planes; Transverse plane slice thickness 10 mm with 0.5-1.0 mm slice gap; coronal plane slice thickness 4–5 mm with 0.5-1.0 mm slice gap	Coronal and axial planes	All	Visual inspection	Qualitative	Presence of signal intensity alterations including muscle oedema, fatty infiltration and abnormal enhancement
Gooding et al., 2016 [29]	Not reported	3T; turbo spin echo; slice thickness 10 mm; 0 mm interslice gap; acquisition time 7 min	From most posterior aspect calcaneus to the toes; for each muscle the series of 3 contiguous slices that provided the largest CSA used for analysis	ABH, FDB, ABDM, QP, FDM, ADDH-O, FHB, Interosseous & lumbricals (together)	Each muscle manually outlined; pixel-by-pixel count based on active range (any pixel that exceeded the lower threshold)	N/A	Percentage increase in muscle activation (pre to post exercise)
Green & Briggs, 2013 [32]	Not reported	Magnet strength not specified; T1- & proton density weighted; slice thickness not specified; interslice gap 5 mm;	Coronal plane images acquired with the plane tilted antero-superiorly to lie perpendicular to the long axis of the second metatarsal	5 groups: (1) Medial - ABH, FHB; (2) ADDH; (3) Central - FDB, QP, lumbricals; (4) Interosseous; (5) Lateral - ABDM, FDM	Measured using a freehand cursor to trace around the muscle groups	N/A	Maximum muscle CSA (cm^2^)
Greenman et al., 2005 [15]	Not reported	3T; T2-weighted H spin echo: thickness 2.5 mm; acquisition time 6 min 24 s; RARE pulse: slice thickness 25 mm; acquisition time 4 min	Axial plane slices; 10 contiguous locations	All, as a single group	Interactive data language software; an outline of the muscle tissue and reference standard was created using a contour mapping function set to a single level that was equal to the noise threshold value; count of pixels that represented signal from foot tissues	N/A	Muscle area- to -total area ratio
Kurihara et al., 2014 [33]	Not reported	1.5T; T1-weighted fast spin echo; slice thickness 4 mm; acquisition time ~ 9 min	Whole foot (sesamoids to calcaneal tuberosity); contiguous slices; acquired perpendicular to plantar aspect of foot; image at the MTP joint that was near 20% longitudinal foot length was selected for analysis	3 muscle groups: 1) Medial – FHB, FDB, QP, ABH and lumbricals; 2) ADDH; 3) Lateral – ABDM, FDB, interosseus	Manual; SliceOmatic software; excluded non-contractile tissues where possible	N/A	Muscle CSA (cm^2)^
Lin et al., 2016 [16]	Not reported	3T; T1-weighted ^1^H spin: slice thickness 2.5 mm; acquisition time 6 min 24 s; ^31^P-RARE: slice thickness 25 mm; scan time 4 min	Acquired in a plane perpendicular to longitudinal direction of foot through the metatarsal head region; 10 contiguous; Selected for analysis: level of the 5th metatarsal head from T1-weighted images	3 muscle regions: (1) FHB medial head; (2) ADDH, FHB lateral head, lumbricals; (3) Interosseous, FDM, ABDM	Manually outlined using tracing tool in OsiriX software; pixel threshold technique	Semi-quantitative 5-point scale	Ratio (^31^P/^1^H) of the area of viable muscle tissue to total area outlined; Fatty atrophy: 0 = healthy muscle with no atrophy; 1 = mild atrophy; 2 = moderate atrophy; 3 = severe atrophy; 4 = almost no or no muscle tissue visible.
Miller et al., 2014 [27]	Intra-rater reliability; five measurements on each muscle over multiple days; mean measurement relative error 0.2–4.3%	1.5T; T2 turbo spin echo fat saturation; slice thickness 5 mm	Coronal, sagittal, axial scans; entire foot; muscle measurement from axial scan	ABH; FDB; ABDM	Manually traced in ImageJ	N/A	Muscle CSA (mm^2^) Muscle volume (mm^3^) *log normalised to foot length*
Pelayo-Negro et al., 2014 [22]	Not reported	1.5T; T1-weighted fast spin-echo; fat suppressed proton density-T2-weighted fast spin-echo	Slice thickness not specified; axial and coronal planes	All, as a single group	Visual inspection	Semi-quantitative; 5-point scale	Fatty infiltration: 0 = no evidence of fatty infiltration; 1 = some fatty streaks; 2 = fat evident but less extensive than muscle; 3 = fat equal to muscle; and stage 4 = fat more extensive than muscle.
Recht et al., 2007 [21]	Not reported	0.2 to 1.5T; T1- and T2-weighted (with or without fat suppression) in coronal; STIR or T2-weighted in sagittal	All images	ABDM	Visual inspection	Semi-quantitative; 4-point scale	Fatty atrophy: grade 0 = no fat or minimal fatty streaks; 1 = increased fat within the muscle but greater amount of muscle; 2 = equal amounts of fat and muscle; 3 = greater amount of fat than muscle
Savnik et al., 2000 [31]	Not reported	1.5T; T1-weighted spin echo; T2-weighted spin echo and STIR; slice thickness 3–4 mm; interslice gap 0.3–0.4 mm	Sagittal images acquired; coronal reformatted images used for measurement	QP; FDB; EH	Measured using region-of-interest area function	N/A	Muscle largest diameter and transverse area
Schmid et al., 2009 [20]	Intra-rater reliability CSA measures; 20% of measures repeated; ICCs > 0.9 Inter-rater reliability fatty atrophy score; kappa 0.33 to 0.68	1.5T; T1- and T2-weighted; Slice thickness 3-3.5 mm	Coronal images; CSA measures at the level where the bony insertion of the tibiocalcaneal ligament at the calcaneus was best visualised	ABDM; FDB; ABH; QP	Visual inspection and measurements using OsiriX software	Semi-quantitative 3-point scale	Muscle CSA (cm^2^) Fatty muscle atrophy: 0 = normal muscle; 1 = mild fatty atrophy with more muscle than fat; 2 = substantial fatty atrophy with more fat than muscle or equal parts fat and muscle
Severinsen et al., 2007 [17]	Not reported	1.5T; T1 spin echo; slice thickness 1.5 mm; inter-slice interval 10 mm	The first section being randomly placed within the first interslice interval	All, as a single group	Semi-automated user set signal intensity threshold, stereological point-counting technique	N/A	Muscle volume (mm^3^)
Taddei et al., 2018 [28]	Not reported	1.5T; T1-weighted spin-echo; slice thickness 4 mm; contiguous	Images acquired perpendicular to the plantar aspect of the foot; between the most proximal and most distal images in which every intrinsic foot muscle is visible	ABH; ABDM; FHB; FDB	Measured by ImageJ planimeter software for each muscle at each slice	N/A	Muscle CSA (mm^2^)
Taddei et al., 2020 [30]	Not reported	1.5T; T1-weighted spin-echo; slice thickness 4 mm; contiguous	Images acquired perpendicular to the plantar aspect of the foot; between the most proximal and most distal images in which every intrinsic foot muscle is visible	ABH; ABDM; FHB; FDB	Measured by ImageJ planimeter software for each muscle at each slice	N/A	Muscle CSA (mm^2^) Muscle volume (cm^2^)

*ABH* Abductor hallucis, *ABDM* Abductor digiti minimi, *ADDH* Adductor hallucis (oblique and transverse heads combined), *ADDH-O* Adductor hallucis oblique head, *ADDH-T* Adductor hallucis transverse head, *EDB* Extensor digitorum brevis, *EH* Extensor hallucis, *FDB* Flexor digitorum brevis, *FDM* Flexor digiti minimi, *FHB* Flexor hallucis brevis, *QP* Quadratus plantae, *CSA* Cross-sectional area, *N/A* Not applicable

Of studies of intrinsic foot muscle morphology (i.e. volume, cross-sectional area and/or thickness), some have measured the intrinsic foot muscles as a group (i.e. total contractile tissue volume) [7, 8, 12–15, 17–19, 26], whereas others report individual muscles (e.g. abductor hallucis, flexor hallucis brevis, etc.) [20, 24, 25, 27–30, 31] or muscle regions (e.g. muscle tissue under the first metatarsal head, medial/central/lateral muscle groups) [9, 16, 32, 33]. Few studies have evaluated intrinsic foot muscle composition [10, 11, 13, 14, 16, 20–23] and most have used qualitative grading scales of fatty atrophy, such as the five-point Goutellier scale [10, 11, 16, 20–22]. Only two studies [13, 14] have quantified intermuscular adipose tissue volume, however this was performed for the intrinsic foot muscles as a whole, and not individual muscles. Low image resolution has limited the accuracy of segmentation of the architecturally complex intrinsic foot muscle group [12, 29, 32, 33].

Advanced MRI technologies and techniques, such as ultra-high-field scanners and chemical-shift sequences, offer the opportunity to enhance current evaluation and understanding of intrinsic foot muscle morphology. With double the signal-to-noise-ratio of 3T, imaging at 7T offers greater precision and accuracy towards quantification of intrinsic foot muscle morphology, particularly the segmentation of individual muscles and has not yet been realised. Chemical-shift MRI (e.g. DIXON [Siemens], IDEAL [General Electric], mDIXON [Phillips], FatSep™ [Hitachi], or WFS [Toshiba]) produces water-only and fat-only images from dual-echo and/or multi-echo acquisitions, which permits accurate quantification of muscle composition (e.g. percentage muscle fat infiltration). The aim of this paper is to provide a proof-of-concept method for the measurement of intrinsic foot muscle morphology and composition with high-field MRI. As part of a series of papers [34, 35], here we also aim to promote improved reporting of image acquisition and image measurement procedures.

## Methods

**Fig. 1:**
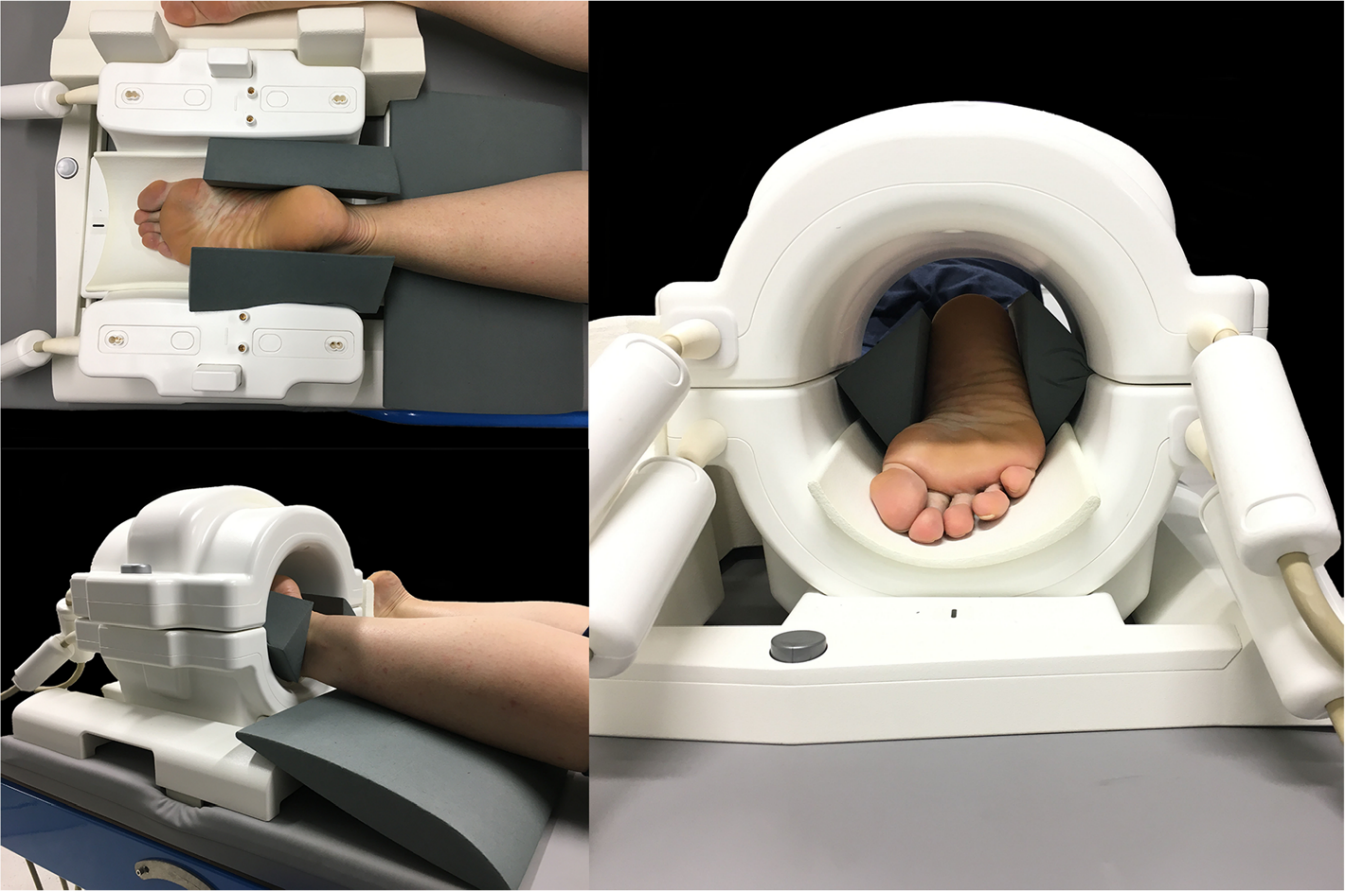
Participant position for 7T and 3T scanners

One healthy female (39 years, mass 65 kg, height 1.73 m, body mass index 21.7, foot length 26.2 cm) was recruited by convenience to participate in the proof-of-concept study. Ethical approval was provided by The University of Queensland Human Research Ethics Committee (#2,018,001,150). The participant provided informed written consent and attended The University of Queensland’s Centre for Advanced Imaging for a single imaging session. The participant underwent MRI of the whole foot on two different magnetic resonance scanners: a 7T MAGNETOM (Siemens AG, Erlangen, Germany) magnetic resonance scanner with a knee coil (QED knee coil 1TX/28Rx) and a 3T MAGNETOM Prisma (Siemens AG, Erlangen, Germany) magnetic resonance scanner with a knee coil (Tx/Rx 15-channel knee coil). Patient positioning was standardised for both scanners (Fig. 1). To enable positioning of the foot within the coil, and allow maximal visualisation of the foot on the MR images, the participant was positioned in prone with the knee supported in slight flexion by a foam pad under the tibia, and the ankle and foot supported in plantar flexion within the receiver coil. Foam padding was placed between the dorsum of the foot and the receiver coil. The position of the foot and ankle was supported by foam wedges and sandbags to minimise movement.

A T1-weighted VIBE – radio-frequency spoiled 3D steady state GRE – sequence of the whole foot was acquired on each scanner. For comparability, acquisition time was standardised (7T: 4 minutes 50 seconds; 3T: 4 minutes 47 seconds). 7T acquisition parameters were: repetition time 11 ms; echo time 2.04 ms; flip angle 3 degrees; field of view 175 × 224 mm^2^; acquired voxel dimensions 0.50 × 0.50 mm^2^; 0.50 mm contiguous slices; 176 slices; Bandwidth 429 Hz/Px). 3T acquisition parameters were: repetition time 15 ms; echo time 2.45 ms; flip angle 10 degrees; field of view 220 × 220 mm^2^; acquired voxel dimensions 0.60 × 0.60 mm^2^; 0.60 mm contiguous slices; 144 slices; Bandwidth 430 Hz/Px). To demonstrate the full capability of the 7T scanner, a high-resolution fat/water separation image was also acquired using a 3D 2-point DIXON sequence (repetition time 11 ms; echo time 3.06 ms and 5.61 ms; flip angle 3 degrees; field of view 111 × 223 mm^2^; acquired voxel dimensions 0.38 × 0.38 mm^2^; 0.38 mm contiguous slices; 256 slices; bandwidth 434 Hz/Px; acquisition time 11 min 12 s).

**Fig. 2:**
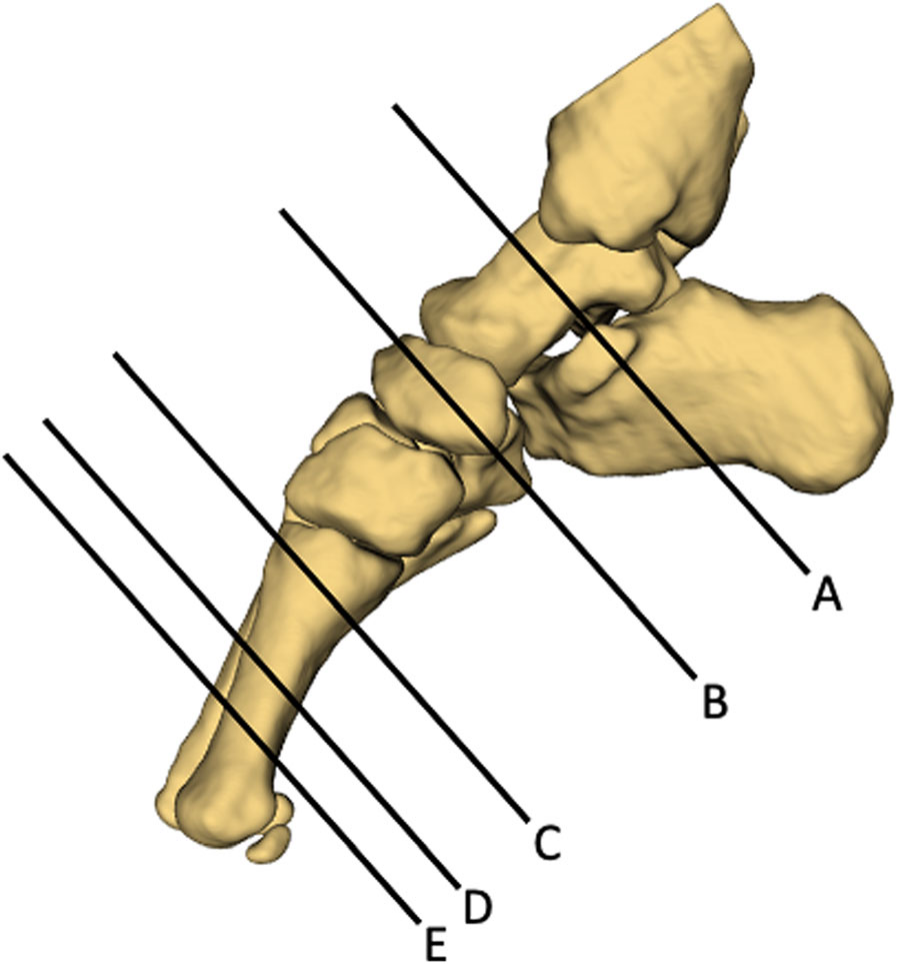
Image locations: (A) the level of the sustentaculum tali, (B) immediately distal to the talo-navicular joint, (C) immediately distal to the first tarso-metatarsal joints, (D) mid-shaft of the first metatarsal, and (E) distal shaft of the first metatarsal (prior to the metatarsal head)

For comparison of 3T and 7T image resolution, images were imported to OsiriX MD (Pixmeo SARL, Switzerland) and coronal plane images from each T1-weighted series were selected at five anatomical locations (Fig. 2): (A) the level of the sustentaculum tali, (B) immediately distal to the talo-navicular joint, (C) immediately distal to the first tarso-metatarsal joints, (D) the mid-shaft of the first metatarsal and (E) the distal shaft of the first metatarsal (up to the metatarsal head). These locations were selected to provide example images from the rearfoot, midfoot and forefoot regions, as well as to include visualisation of all intrinsic foot muscles.

For the quantitative assessment of intrinsic foot muscle structure and composition, muscle volume and percentage muscle fat infiltration were measured from the 7T fat-water images using 3D Slicer software [36]. Regions of interest were manually contoured on each slice for each muscle (abductor hallucis, adductor hallucis, flexor digitorum brevis, quadratus plantae, abductor digiti minimi, flexor hallucis brevis, flexor digiti minimi, lumbricals, and plantar and dorsal interossei). Muscle volumes and the percentage of muscle fat infiltration (muscle fat infiltration % = Fat/(Fat + Water) x 100) were calculated from the segmented images for each muscle using established methods [37, 38]. For illustrative purposes only (viewing the three-dimensional reconstruction), the bones of the leg and foot were also segmented on images.

## Results

**Fig. 3:**
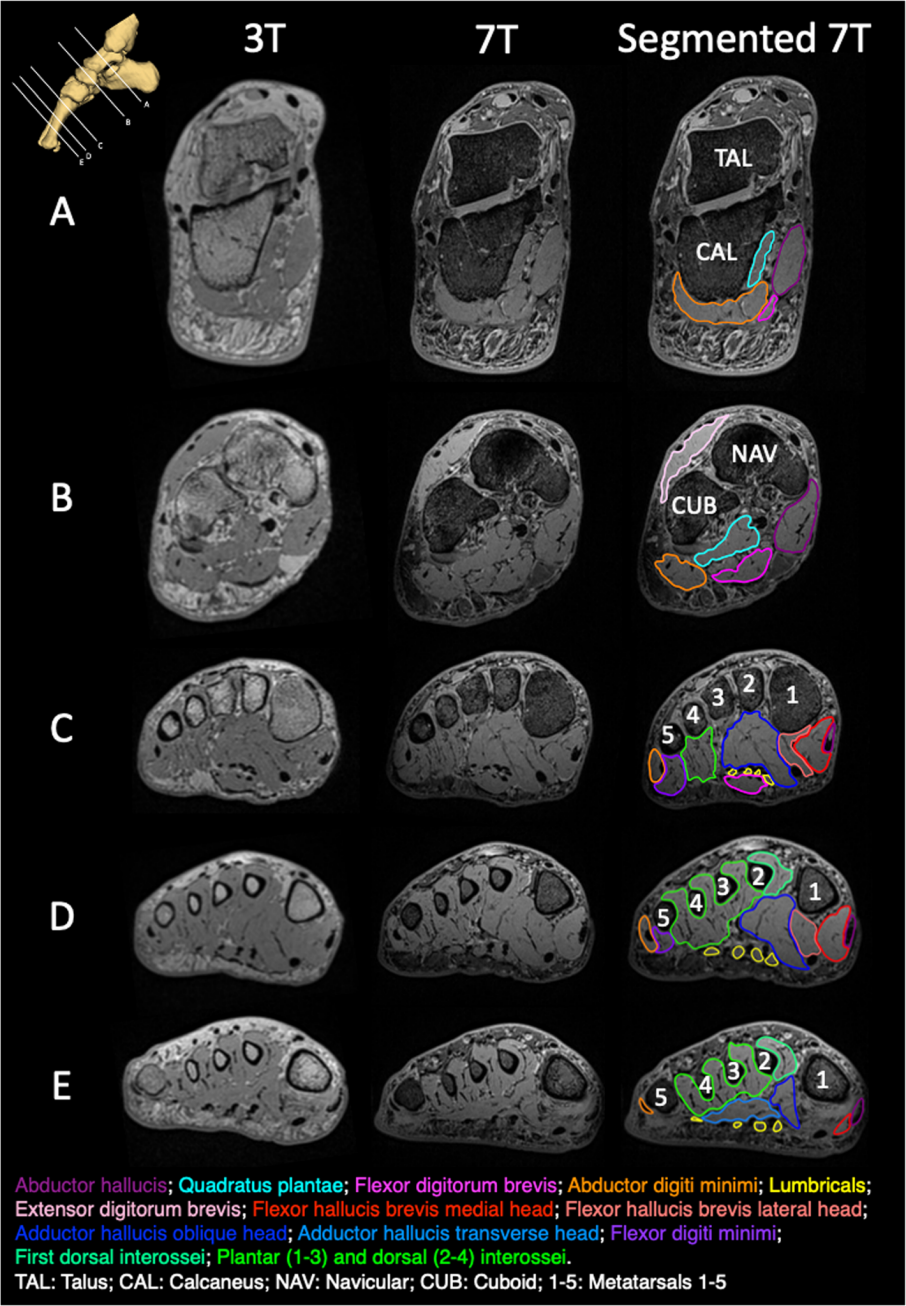
Example T1-weighted images from 3T and 7T scanners, with muscle segmentations illustrated on the 7T images

Figure 3 presents the coronal plane images at each of the five locations for the 3T and 7T sequences. For reference, Fig. 3 also displays the region of interest (segmentation) for each muscle on the 7T images. As demonstrated, 7T produced higher image resolution, which enhanced the visualisation of individual muscle borders for manual segmentation. At the rearfoot (Fig. 3a), the abductor hallucis was easily visualised on both 3T and 7T images, but 7T was required to visualise muscle borders between quadratus plantae and abductor digiti minimi, as well as between abductor digiti minimi and flexor digitorum brevis. At the midfoot (Fig. 3b), although individual muscle borders were enhanced on 7T images, muscle borders of extensor digitorum brevis, abductor hallucis, flexor digitorum brevis, quadratus plantae, abductor digiti minimi were visible on both 3T and 7T images. At the forefoot (Fig. 3c, d and e), the enhanced visualisation of individual muscle borders on 7T was required to distinguish flexor digiti minimi from abductor digiti minimi, adductor hallucis from flexor hallucis brevis, and abductor hallucis from flexor hallucis brevis. 7T images also provided sufficient resolution to visualise and individually segment discrete portions of some muscles, for example, the transverse and oblique portions of the adductor hallucis (Fig. 3E), as well as the medial and lateral portions of the flexor hallucis brevis (Fig. 3c, d). The muscle border of the first dorsal interossei was visible on 7T and could be segmented individually, however differentiation of the other individual plantar and dorsal interossei was challenging, even on 7T images. Consequently, these muscles were segmented as a group (Fig. 3c, d, e).

**Fig. 4:**
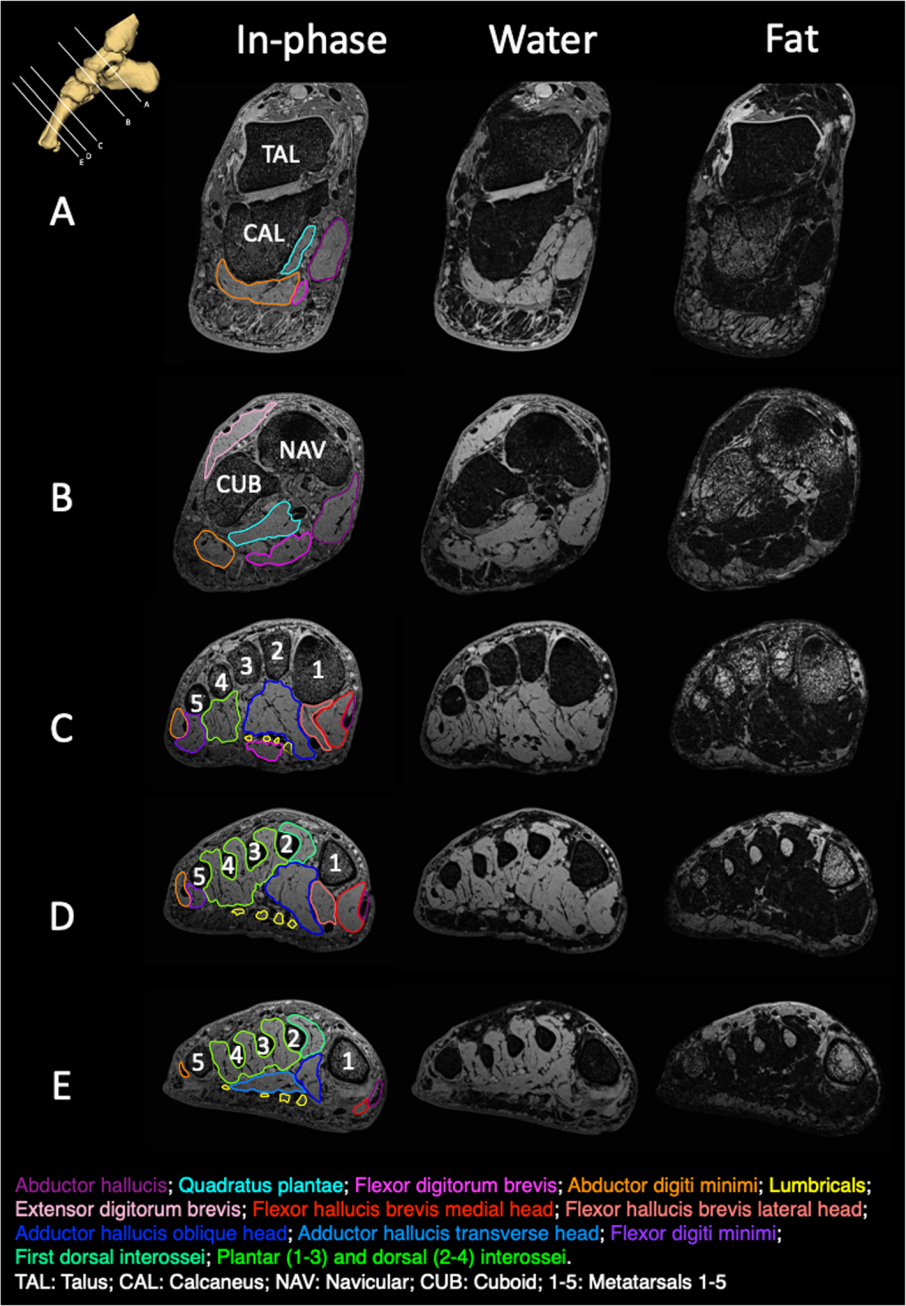
Example fat-water images from 7T scanner, with muscle segmentations illustrated on the in-phase images

**Fig. 5:**
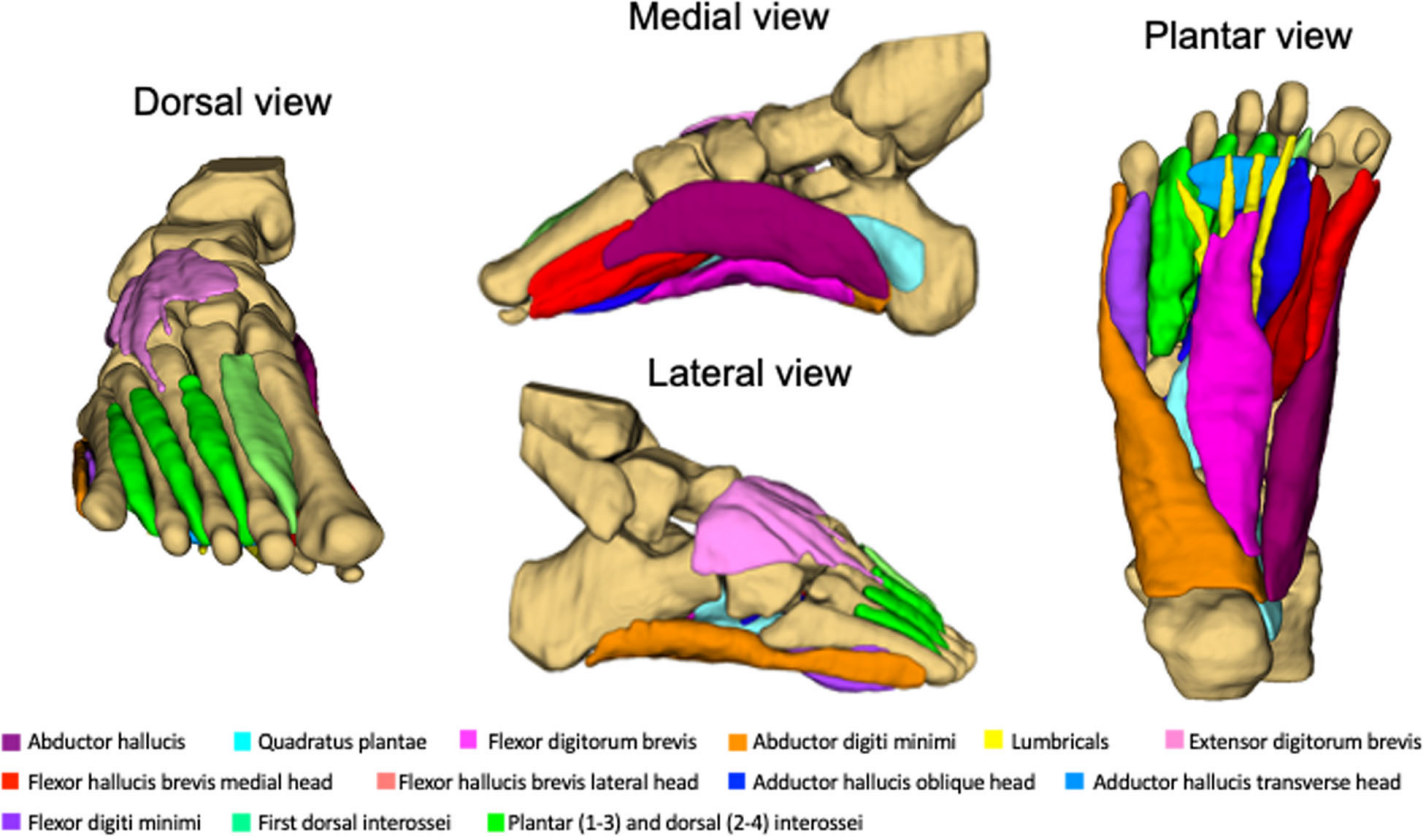
Three-dimensional reconstruction of the intrinsic foot muscles from segmentations performed in 3D Slicer

Figure 4 illustrates fat and water images, in the coronal plane, from the same five anatomical locations (Fig. 2). For reference, the in-phase image is also included with the region of interest (segmentation) displayed for each muscle. Measurements of muscle volume and percentage muscle fat infiltration are provided in Table 2. Muscle volume ranged from 1.5 cm^3^ (lumbricals) to 19.8 cm^3^ (dorsal and plantar interossei) and percentage muscle fat infiltration ranged from 9.2% (extensor digitorum brevis) to 15.0% (abductor digiti minimi). Figure 5 displays a three-dimensional reconstruction of the intrinsic foot muscles from 3D Slicer.

**Table 2:** Muscle volume and percentage muscle fat infiltration (MFI)

	Volume (cm^3^)	% MFI
Abductor hallucis	17.4	11.3
Quadratus plantae	13.9	12.6
Flexor digitorum brevis	10.2	12.4
Abductor digiti minimi	17.3	15.0
Flexor hallucis brevis		
* Medial head*	7.8	10.8
* Lateral head*	5.9	11.3
Adductor hallucis		
* Oblique head*	17.8	11.0
* Transverse head*	1.8	14.0
Flexor digitorum brevis	4.4	13.5
Lumbricals	1.5	12.2
Interosseous		
* First dorsal interosseous*	4.0	13.1
* Plantar interosseous (1–3) and dorsal interosseous (2–4)*	19.8	12.8
Extensor digitorum brevis	7.7	9.2

## Discussion

Ultra-high-field (7T) MRI enabled the establishment of quantitative methods to provide more detailed measures of intrinsic foot muscle morphology (volume) and composition (percentage muscle fat infiltration) than has been possible with previous technology. In this proof-of-concept study, we compared images acquired on 7T and 3T scanners, with similar acquisition times, in a single participant on the same day. As 7T MRI has double the signal-to-noise ratio of 3T, the image contrast was, as expected, superior at 7T scanning. The higher resolution was required to enable clear visualisation of anatomical borders for segmentation of individual muscles, particularly in the forefoot. Our observation is supported by previous reports from several authors that segmenting individual intrinsic foot muscles was challenging at lower field strength due to lower image quality [12, 29, 32], as well as the wider literature on morphological musculoskeletal imaging that reports increased spatial resolution at 7T [39].

Although the resolution was lower for the images at 3T than 7T, the lower resolution might be sufficient to enable the segmentation and measurement of cross-sectional area of some muscles at some locations. For example, abductor hallucis at the rearfoot and midfoot, and extensor digitorum brevis, flexor digitorum brevis, quadratus plantae and abductor digiti minimi at the midfoot, were visible on the 3T images. Previous investigations have reported measurements of cross-sectional area of these muscles of the rearfoot using lower field strengths (1.5T) [20, 31]. However, our findings, and reports from others (using lower magnetic field strengths) [12, 29, 32, 33] of the difficulty to clearly visualise borders of the smaller muscles in the forefoot, draws into question the clinical meaningfulness of individual muscle volumes previously reported from 3T or lower field scanners [24, 25, 27, 28, 30]. For future studies using magnetic field strengths of 3T or less, we caution the segmentation of individual intrinsic foot muscles in the forefoot, and recommend that 7T MRI is used to ensure segmentation precision in that regard. Alternatively, muscle volumes could be reported for muscle groups (e.g. abductor hallucis together with flexor hallucis brevis, abductor digiti minimi together with flexor digiti minimi) [16, 32], or the intrinsic foot muscles as a whole [7, 8, 13, 14, 17–19, 26], similar to that reported by previous studies.

In this report of a new method, we follow suggestions from the earlier papers in this series [34, 35] towards quantification of muscle fat infiltration for individual intrinsic foot muscles. Using semi-quantitative methods, previous MRI studies at the foot have reported low fatty infiltration in healthy participants [11, 16, 20]. One study that used a quantitative method [13, 14], reported that 22.7% of total intrinsic foot muscle volume was intramuscular adipose tissue (muscle fat infiltration) in 12 healthy adults (mean age 57 years). This was evaluated for the intrinsic foot muscles as a group, not individual muscles. At other areas of the body, muscle fat infiltration has been observed to differ between individual muscles, within a muscle group, in patient populations [40, 41]. Considering different pain, injury and disease conditions of the foot may affect individual muscles differently, individual muscle segmentation would provide more specific information. We recommend that future studies perform individual muscle segmentations when evaluating muscle fat infiltration, however we recognise this may be limited by availability of ultra-high-field MRI (e.g. 7T).

Although the benefits obtained from 7T compared to 3T for musculoskeletal imaging, such as faster scan time (by a mean factor of 2) and increased spatial resolution (2 to 4-fold), have been reported [39], the availability of ultra-high-field MRI is a consideration for research or clinical utility. Availability may be influenced by several factors including cost and patient safety. In 2015 the estimated cost to install a 7T system in the US was approximately $10 million, although cost is expected to go down with widespread adoption of systems by institutions/clinics [42]. The field of ultra-high-field imaging is rapidly evolving with growth from approximately 40 scanners worldwide in 2014 [43] to over 80 scanners worldwide in 2019 [44]. This evolution also includes transition from use primarily for scientific and medical research to the first clinical use at the Mayo Clinic Rochester in 2017, as well as the number of scanners available in the marketplace. User costs are also a consideration for researchers and patients. To provide context, at our institution 7T scan costs are 30–40% higher than 3T for the same scanning time period (e.g. 30 minute scan). In terms of patient safety, relatively few biomedical implants have undergone proper testing at 7T and therefore to mitigate risks related to increased forces on metallic implants and the unpredictable radiofrequency-induced tissue heating with ultra-high-field strengths, a significant portion of research participants/patients may be precluded from currently undergoing 7T imaging [45].

Operational parameters can influence the quality of images that are acquired from MRI. As is evident in Table 1, a wide range of parameters have been used to evaluate intrinsic foot muscle morphology in previous work and this has been reported with variation in detail. To enable comparisons between foot imaging studies in future, standardised reporting of parameters is required. We propose reporting of the following operation parameters: field strength (e.g. 7-Tesla); sequence type (e.g. 2-point DIXON (3D fast-field echo T1) whole foot); repetition time (e.g. TR 11 ms); echo time (e.g. TE 3.06 ms and 5.61 ms); flip angle (e.g. 3°); field of view (e.g. FOV 111 × 223 mm^2^); acquired voxel dimensions (e.g. 0.38 × 0.38 mm^2^); reconstructed voxel dimensions (e.g. 0.38 × 0.38 mm^2^); bandwidth (e.g. 434 Hz/Px); acquisition time (e.g. TA 11 min 49 s); slice thickness (e.g. 0.38 mm) and number of sliced (e.g. 640 slices). Additionally, to improve transparency and reproducibility, image measurement methods should be described in detail, with the strong recommendation to include figures to illustrate muscle segmentation.

We report images of the intrinsic foot muscles acquired using 3T and 7T with one healthy individual, towards establishing the feasibility of quantifying intrinsic foot muscle structure and composition using higher-field (7T) MRI. It is our hope that this foundational effort can facilitate future studies using these methods, both in healthy individuals and in those with lower limb pain, injury or disease, in order to: (i) enhance understanding of structure and compositional changes in the intrinsic foot muscles across different populations and (ii) generate a body of literature that can be pooled and further scrutinised in systematic reviews with meta-analyses. Although our data illustrates the difference in image quality between 7T and 3T scanners, further research is required to assess the influence of field strength on measurement properties, such as the repeatability of measurements, an aspect that is lacking from the existing literature (see Table 1). Validity of measures should be considered by comparison with anatomical atlases from cadaveric tissue; as reported in the previous methods papers [34, 35]. As manual segmentation of the individual intrinsic foot muscles is time consuming (approximately 6 hours per foot for our data) and rater-dependent, development of automated methods may improve the efficiency and objectivity of muscle measures. Deep learning convolutional neural network models for muscle segmentation and automatic muscle fat infiltration calculation using fat-water images have demonstrated high test reliability and accuracy for muscles in other body regions [46]. This is an area that should be explored in future studies, as it may improve the ability to quantify and monitor foot muscle structure and composition in individuals with lower limb pain, injury or disease; saving time from arduous manual segmentation techniques, and increase likelihood of clinical implementation.

## Conclusions

We have used ultra-high-field (7T) MRI to establish a method to quantify muscle morphology and composition of individual intrinsic foot muscles. This method can be used in future studies to better understand intrinsic foot muscle morphology and composition in healthy individuals, as well as those with lower limb disorders. Following on from papers at the cervical and lumbar spine [34, 35], we also emphasise that improved reporting of image acquisition parameters and image measurement procedures is required to assist consistency and allow accurate comparison between studies.

## Data Availability

All of the data supporting the findings are contained within the manuscript.

## Abbreviations

MRI: Magnetic resonance imaging
T: Tesla
TE: Echo time
TR: Repetition time
US: Ultrasound imaging

## References

[1] Kelly LA, Farris DJ, Cresswell AG, Lichtwark GA (2019). Intrinsic foot muscles contribute to elastic energy storage and return in the human foot. J Applied Physiol.

[2] Farris DJ, Kelly LA, Cresswell AG, Lichtwark GA (2019). The functional importance of human foot muscles for bipedal locomotion. P Natl Acad Sci USA.

[3] Riddick R, Farris DJ, Kelly LA (2019). The foot is more than a spring: human foot muscles perform work to adapt to the energetic requirements of locomotion. J R Soc Interface.

[4] Soysa A, Hiller C, Refshauge K, Burns J (2012). Importance and challenges of measuring intrinsic foot muscle strength. J Foot Ankle Res.

[5] Lieber RL, Friden J. Clinical significance of skeletal muscle architecture. Clin Orthop Relat R. 2001;383:140–51. 10.1097/00003086-200102000-00016 11210948

[6] McGregor RA, Cameron-Smith D, Poppitt SD (2014). It is not just muscle mass: a review of muscle quality, composition and metabolism during ageing as determinants of muscle function and mobility in later life. Longev Healthspan.

[7] Andersen H, Gjerstad MD, Jakobsen J (2004). Atrophy of foot muscles: a measure of diabetic neuropathy. Diabetes Care.

[8] Andreassen CS, Jakobsen J, Ringgaard S, Ejskjaer N, Andersen H (2009). Accelerated atrophy of lower leg and foot muscles–a follow-up study of long-term diabetic polyneuropathy using magnetic resonance imaging (MRI). Diabetologia.

[9] Brash PD, Foster J, Vennart W, Anthony P, Tooke JE (1999). Magnetic resonance imaging techniques demonstrate soft tissue damage in the diabetic foot. Diabet Med.

[10] Bus SA, Maas M, Lindeboom R (2006). Reproducibility of foot structure measurements in neuropathic diabetic patients using magnetic resonance imaging. J Magn Reson Imaging.

[11] Bus SA, Maas M, Michels RPJ, Levi M (2009). Role of intrinsic muscle atrophy in the etiology of claw toe deformity in diabetic neuropathy may not be as straightforward as widely believed. Diabetes Care.

[12] Bus SA, Yang QX, Wang JH, Smith MB, Wunderlich R, Cavanagh PR (2002). Intrinsic muscle atrophy and toe deformity in the diabetic neuropathic foot: a magnetic resonance imaging study. Diabetes Care.

[13] Cheuy VA, Commean PK, Hastings MK, Mueller MJ (2013). Reliability and validity of a MR-based volumetric analysis of the intrinsic foot muscles. J Magn Reson Imaging.

[14] Cheuy VA, Hastings MK, Commean PK, Ward SR, Mueller MJ (2013). Intrinsic foot muscle deterioration is associated with metatarsophalangeal joint angle in people with diabetes and neuropathy. Clin Biomech.

[15] Greenman RL, Khaodhiar L, Lima C, Dinh T, Giurini JM, Veves A (2005). Foot small muscle atrophy is present before the detection of clinical neuropathy. Diabetes Care.

[16] Lin YC, Wu J, Baltzis D, Veves A, Greenman R (2016). MRI assessment of regional differences in phosphorus-31 metabolism and morphological abnormalities of the foot muscles in diabetes. J Magn Reson Imaging.

[17] Severinsen K, Obel A, Jakobsen J, Andersen H (2007). Atrophy of foot muscles in diabetic patients can be detected with ultrasonography. Diabetes Care.

[18] Chang R, Kent-Braun JA, Hamill J (2012). Use of MRI for volume estimation of tibialis posterior and plantar intrinsic foot muscles in healthy and chronic plantar fasciitis limbs. Clin Biomech.

[19] Cheung RT, Sze LK, Mok NW, Ng GY (2016). Intrinsic foot muscle volume in experienced runners with and without chronic plantar fasciitis. J Sci Med Sport.

[20] Schmid DT, Hodler J, Mengiardi B, Pfirrmann CW, Espinosa N, Zanetti M (2009). Fatty muscle atrophy: prevalence in the hindfoot muscles on MR images of asymptomatic volunteers and patients with foot pain. Radiology.

[21] Recht MP, Grooff P, Ilaslan H, Recht HS, Sferra J, Donley BG (2007). Selective atrophy of the abductor digiti quinti: an MRI study. Am J Roentgenol.

[22] Pelayo-Negro AL, Gallardo E, Garcia A, Sanchez-Juan P, Infante J, Berciano J (2014). Evolution of Charcot-Marie-Tooth disease type 1A duplication: a 2-year clinico-electrophysiological and lower-limb muscle MRI longitudinal study. J Neurol.

[23] Gallardo E, Garca A, Combarros O, Berciano J (2006). CharcotMarieTooth disease type 1A duplication: spectrum of clinical and magnetic resonance imaging features in leg and foot muscles. Brain.

[24] Feger MA, Donovan L, Herb CC, Handsfield GG, Blemker SS, Hart JM (2019). Impairment-Based Rehabilitation Increases Lower Leg Muscle Volumes and Strength in Chronic Ankle Instability Patients: A Preliminary Study. J Sport Rehabil.

[25] Feger MA, Snell S, Handsfield GG, Blemker SS, Wombacher E, Fry R (2016). Diminished Foot and Ankle Muscle Volumes in Young Adults With Chronic Ankle Instability. Orthop J Sports Med.

[26] Chen TL-W, Sze LKY, Davis IS, Cheung RTH (2016). Effects of training in minimalist shoes on the intrinsic and extrinsic foot muscle volume. Clin Biomech.

[27] Miller EE, Whitcome KK, Lieberman DE, Norton HL, Dyer RE (2014). The effect of minimal shoes on arch structure and intrinsic foot muscle strength. J Sport Health Sci.

[28] Taddei UT, Matias AB, Ribeiro FIA, Inoue RS, Bus SA, Sacco ICN. Effects of a therapeutic foot exercise program on injury incidence, foot functionality and biomechanics in long-distance runners: Feasibility study for a randomized controlled trial. Phys Ther Sport. 2018;34:216–26. 10.1016/j.ptsp.2018.10.015 30388670

[29] Gooding TM, Feger MA, Hart JM, Hertel J (2016). Intrinsic Foot Muscle Activation During Specific Exercises: A T2 Time Magnetic Resonance Imaging Study. J Athl Train.

[30] Taddei UT, Matias AB, Ribeiro FIA, Bus SA, Sacco ICN (2020). Effects of a foot strengthening program on foot muscle morphology and running mechanics: A proof-of-concept, single-blind randomized controlled trial. Phys Ther Sport.

[31] Savnik A, Amris K, Røgind H, Prip K, Danneskiold-Samsøe B, Bojsen-Møller F (2000). MRI of the plantar structures of the foot after falanga torture. Eur Radiol.

[32] Green SM, Briggs PJ (2013). Flexion strength of the toes in the normal foot. An evaluation using magnetic resonance imaging. Foot.

[33] Kurihara T, Yamauchi J, Otsuka M, Tottori N, Hashimoto T, Isaka T (2014). Maximum toe flexor muscle strength and quantitative analysis of human plantar intrinsic and extrinsic muscles by a magnetic resonance imaging technique. J Foot Ankle Res.

[34] Crawford RJ, Cornwall J, Abbott R, Elliott JM (2017). Manually defining regions of interest when quantifying paravertebral muscles fatty infiltration from axial magnetic resonance imaging: a proposed method for the lumbar spine with anatomical cross-reference. BMC Musculoskel Dis.

[35] Elliott JM, Cornwall J, Kennedy E, Abbott R, Crawford RJ (2018). Towards defining muscular regions of interest from axial magnetic resonance imaging with anatomical cross-reference: part II - cervical spine musculature. BMC Musculoskel Dis.

[36] Fedorov A, Beichel R, Kalpathy-Cramer J, Finet J, Fillion-Robin J-C, Pujol S (2012). 3D Slicer as an image computing platform for the Quantitative Imaging Network. Magn Reson Imaging.

[37] Khanna R, Saltzman MD, Elliott JM, Hoggarth MA, Marra GM, Omar I, et al. Development of 3D method to assess intramuscular spatial distribution of fat infiltration in patients with rotator cuff tear: reliability and concurrent validity.(Report). BMC Musculoskel Dis. 2019;20(1):295–5. 10.1186/s12891-019-2631-z PMC6587235 31221138

[38] Crawford JR, Volken CT, Ni Mhuiris M, Bow AC, Elliott AJ, Hoggarth AM (2019). Geography of Lumbar Paravertebral Muscle Fatty Infiltration: The Influence of Demographics, Low Back Pain, and Disability. Spine.

[39] Juras V, Mlynarik V, Szomolanyi P, Valkovič L, Trattnig S (2019). Magnetic Resonance Imaging of the Musculoskeletal System at 7T: Morphological Imaging and Beyond. Top Magn Reson Imaging.

[40] Uthaikhup S, Assapun J, Kothan S, Watcharasaksilp K, Elliott JM (2017). Structural changes of the cervical muscles in elder women with cervicogenic headache. Musculoskelet Sci Pract.

[41] Elliott J, Jull G, Noteboom JT, Darnell R, Galloway G, Gibbon WW (2006). Fatty infiltration in the cervical extensor muscles in persistent whiplash-associated disorders: a magnetic resonance imaging analysis. Spine.

[42] Balchandani P, Naidich TP, Ultra-High-Field MR, Neuroimaging (2014). Am J Neuroradiol.

[43] Regatte RR (2014). Why Buy an Expensive ($7 Million) 7T MRI System for Biomedical Research?. J Magn Reson Imaging.

[44] Barisano G, Sepehrband F, Ma S, Jann K, Cabeen R, Wang DJ (2019). Clinical 7 T MRI: Are we there yet? A review about magnetic resonance imaging at ultra-high field. British J Radiol.

[45] Hoff MN, McKinney At, Shellock FG, Rassner U, Gilk T, Watson RE (2019). Safety Considerations of 7-T MRI in Clinical Practice. Radiology.

[46] Weber KA, Smith AC, Wasielewski M, Eghtesad K, Upadhyayula PA, Wintermark M (2019). Deep Learning Convolutional Neural Networks for the Automatic Quantification of Muscle Fat Infiltration Following Whiplash Injury. Sci Rep.

